# Supplementary material for: Cas9-mediated endogenous plasmid loss in Borrelia burgdorferi
Source: PLoS One. 2022 Nov 28;17(11):e0278151. doi: 10.1371/journal.pone.0278151 (PMC9704580; doi:10.1371/journal.pone.0278151)
Supplement: S1 Fig — (PDF) [file pone.0278151.s001.pdf]

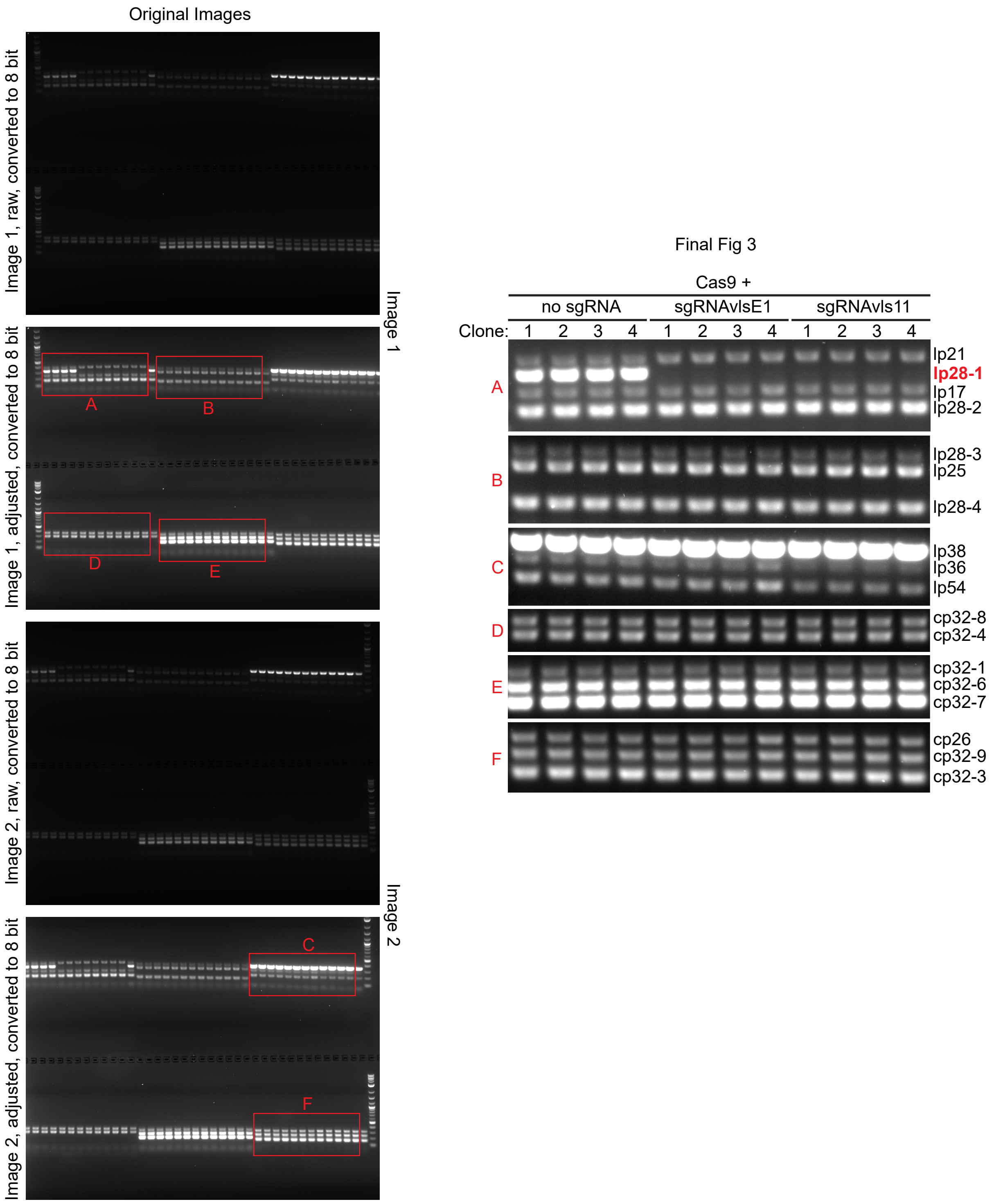

S1 Fig. Original gel images used to generate Fig 3. (Left) The two full gel scans (Images 1 and 2, respectively) were converted to 8-bit format or were brightness-adjusted to emphasize the bands, then converted to 8-bit format. The general areas that were cropped (and slightly rotated in some cases) to generate Fig 3 are highlighted by red rectangles. The rectangles were drawn intentionally larger than the cropped areas so as not to obscure the bands. Correspondence between the cropped areas and Fig 3 (reproduced at Right) is indicated by red capital letters.
