# Supplementary material for: Cas9-mediated endogenous plasmid loss in Borrelia burgdorferi
Source: PLoS One. 2022 Nov 28;17(11):e0278151. doi: 10.1371/journal.pone.0278151 (PMC9704580; doi:10.1371/journal.pone.0278151)
Supplement: S2 Fig — (PDF) [file pone.0278151.s002.pdf]

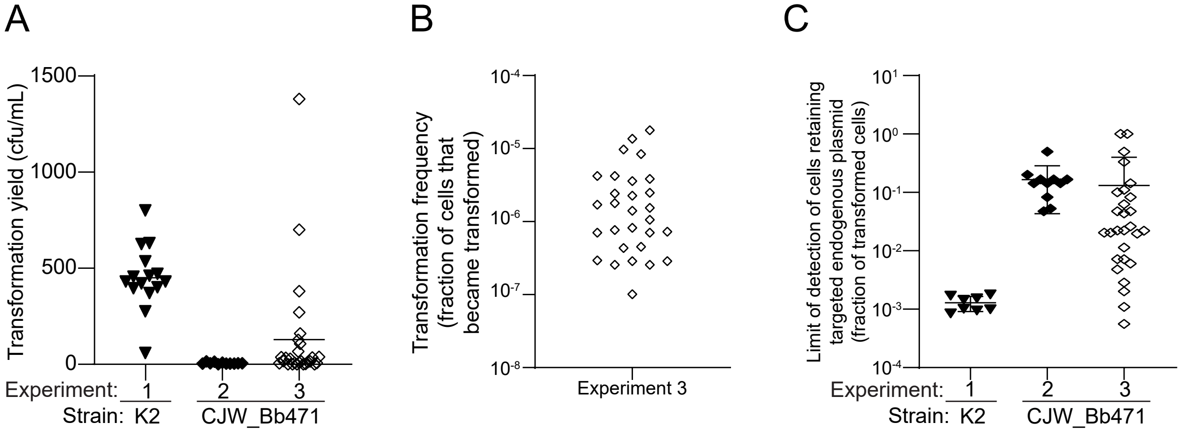

S2 Fig. Transformation statistics. A. Transformation yield, defined as number of shuttle vector-transformed colony forming units (cfu) per mL culture measured by plating under streptomycin selection one day after electroporation. The experiment numbers are the same as in Tables 4 and 5 and are listed at the bottom. Recipient strains are also listed at the bottom. **B.** Plot showing the distribution of transformation frequency values measured in experiment 3 described in (A) and Tables 4 and 5. The transformation frequency is defined as the ratio of the number of cells that got transformed with the Cas9 shuttle vector to the number of cells that survived electroporation. Transformed and viable cell numbers were measured by plating the electroporated cells in the presence or absence of streptomycin. **C.** Plot showing the limit of detection of cells that retained the targeted endogenous plasmid for the individual electroporations performed in experiments 1 through 3 as described in panel (A) and Tables 4 and 5. The limit of detection values were calculated as follows. First, we counted the number of colonies obtained by plating the electroporated *B. burgdorferi* cultures under streptomycin selection and added the values we counted on the plates seeded with the 100, 300, and 900 µL cell culture volumes (1.3 mL total, also see Methods). If plates seeded with larger volumes of culture yielded too many colonies to allow an accurate count, the number of colony-forming cells in 1.3 mL culture was estimated from the number of colonies counted on plates seeded with the lesser culture volume(s). The limit of detection value was then calculated as the inverse of the number of colonies obtained from plating 1.3 mL electroporated culture. Labels are as in (A).
