# Supplementary material for: Cas9-mediated endogenous plasmid loss in Borrelia burgdorferi
Source: PLoS One. 2022 Nov 28;17(11):e0278151. doi: 10.1371/journal.pone.0278151 (PMC9704580; doi:10.1371/journal.pone.0278151)
Supplement: Attachment — Submitted filename: Response to Reviewers.docx [file pone.0278151.s003.docx]

We thank the reviewers for their time and consideration. We are also grateful for their positive comments and valuable input. Please find below, in blue, our point-by-point response to the reviewers’ comments.

Reviewer #1: The manuscript by Takacs et al. describes a CRISPR-Cas9-based method to cure Borrelia burgdorferi (Bb) of some of its many plasmids. The authors build off their prior work using CRISPR-Cas to knock down Bb gene expression. Plasmids that are not required for growth in vitro (but are important in the natural tick-spirochete lifecycle) were successfully cured from Bb by wild type Cas9 nuclease that produces double strand breaks. Plasmids were also targeted by Cas9 point mutants (nickases) that produced single strand lesions on target plasmids.

The genetic tools described by the authors are a welcome addition to the field. The targeted elimination (by wt Cas9) or possible targeted suppression of plasmid copy number or destabilization of plasmids (by Cas9 nickases) will advance the field.

We thank the reviewer for their positive appraisal of our manuscript!

I only have a few minor comments and suggestions for the authors to consider.

1. line 33: Are humans accidental or incidental hosts?

Thank you for flagging this. We clarified that humans are dead-end host, meaning that they don’t contribute to the perpetuation of the transmission cycle.

2. Line 33: Isn’t Bb ‘always’ maintained (as opposed to ‘typically’) through a transmission cycle between vertebrates and ticks?

Thank you for pointing this out. We removed the word “typically”.

3. Line 64: “more targeted approach” seems more appropriate than “easier”

The plasmid exchange method is probably as targeted as our method is. We have revised the sentence to write “A more streamlined method”, which we believe to be correct.

4. Line 68: Maybe I am misunderstanding, but isn’t a double strand break in the chromosome lethal to all (as opposed to several) Bacteria if not repaired?

We meant to say that double strand breaks are not always repaired through homologous recombination. Some bacteria can perform non-homologous end-joining repair of these DNA lesions. We added a statement clarifying this aspect.

5. Line 79: CRISPR-Cas is an adaptive bacterial immune system, not innate

Thank you for flagging this. We removed the “innate” term.

6. Line 204-5: ‘Assays retention’ and ‘examines retention’ was confusing

We replaced the confusing terms with “measures” and “quantifies”.

7. Line 211 and elsewhere: Does plasmid destabilization mean something distinct from plasmid loss? If so, can the authors make this explicit?

No, we meant plasmid loss. This is now clarified.

8. Lines 210-: Please call out the figures showing data that supports claims in each of these sentences.

We called the appropriate Tables and Figures as requested.

9. The methods are very detailed, thank you

Thank you!

10. Can the authors please provide the DNA sequence of the 10,910 base pair regions encompassing vlsE and silent cassettes vls2-vls16 in K2 and B31? These sequences may be useful to labs studying antigenic variation in Bb.

We deposited the K2 *vls* sequence with Genbank and reported the accession number (OP620651, line 192). The B31 *vls* sequence was previously determined and reported by Fraser et al Nature 1997 when the full B31 genome was sequenced. The sequence is available from Genbank under accession number AE000794.2.

Reviewer #2: The authors of this study have developed a method to selectively target individual plasmids of the Lyme disease bacterium. The authors rely on an inducible cas9 based system to nick targeted DNA via sgRNAs, and show that the targeted plasmids have been lost, while retaining the other endogenous plasmids. They demonstrate that this is most efficient with wild-type cas9 that nicks both DNA strands, and less efficient when either of two mutant cas9s that only nick one strand of DNA are used.

This novel method of removing individual plasmids will be broadly useful for researchers studying B. burgdorferi, and the functions that specific plasmids have in culture as well as during the tick-mouse life cycle.

The data are sound, and the manuscript should be accepted as is.

We thank the reviewer for their support!
